# Determinants of non-alcoholic fatty liver disease in young people: Maternal, neonatal, and adolescent factors

**DOI:** 10.1371/journal.pone.0298800

**Published:** 2024-02-22

**Authors:** Johannes Nairz, Alex Messner, Sophia J. Kiechl, Bernhard Winder, Christoph Hochmayr, Alexander E. Egger, Andrea Griesmacher, Ralf Geiger, Elke Griesmaier, Raimund Pechlaner, Michael Knoflach, Ursula Kiechl-Kohlendorfer, the Early Vascular Ageing in the YOUth (EVA4YOU) Study Group

**Affiliations:** 1 VASCage Research Centre on Vascular Ageing and Stroke, Innsbruck, Tyrol, Austria; 2 Department of Pediatrics II, Medical University of Innsbruck, Innsbruck, Tyrol, Austria; 3 Department of Pediatrics III, Medical University of Innsbruck, Innsbruck, Tyrol, Austria; 4 Department of Neurology, Hochzirl Hospital, Zirl, Tyrol, Austria; 5 Department of Vascular Surgery, Feldkirch Hospital, Feldkirch, Vorarlberg, Austria; 6 Central Institute of Medical and Chemical Laboratory Diagnostics (ZIMCL), University Hospital of Innsbruck, Innsbruck, Tyrol, Austria; 7 Department of Neurology, Medical University of Innsbruck, Innsbruck, Tyrol, Austria; The Second Xiangya Hospital of Central South University, CHINA

## Abstract

**Aim:**

To assess the impact of maternal, neonatal, and adolescent factors on the development of non-alcoholic fatty liver disease (NAFLD) in a cohort of 14- to 19-year-old adolescents.

**Methods:**

This study is part of the Early Vascular Ageing in the YOUth study, a single-center cross-sectional study conducted in western Austria. Maternal and neonatal factors were extracted from the mother-child booklet, adolescent factors were evaluated by a face-to-face interview, physical examination, and fasting blood analyses. Liver fat content was assessed by controlled attenuation parameter (CAP) using signals acquired by FibroScan^®^ (Echosense, Paris, France). The association of maternal, neonatal, and adolescent factors with CAP values was analyzed using linear regression models.

**Results:**

In total, 595 adolescents (27.2% male) aged 17.0 ± 1.3 years were included. 4.9% (n = 29) showed manifest NAFLD with CAP values above the 90^th^ percentile. Male sex (p < 0.001), adolescent triglyceride levels (p = 0.021), Homeostatic Model Assessment for Insulin Resistance index and BMI z-score (p < 0.001, each) showed a significant association with liver fat content in the multivariable analysis. Maternal pre-pregnancy BMI was associated with CAP values after adjustment for sex, age, and birth weight for gestational age (p < 0.001), but this association was predominantly mediated by adolescent BMI (indirect effect b = 1.18, 95% CI [0.69, 1.77]).

**Conclusion:**

Components of the metabolic syndrome were the most important predictors of adolescent liver fat content. Therefore, prevention of NAFLD should focus on lifestyle modification in childhood and adolescence.

## Introduction

Non-alcoholic fatty liver disease (NAFLD) is one of the most common chronic liver diseases in children in the developed world [1, 2]. It is defined as fat accumulation in the liver in the absence of excessive alcohol consumption (< 140 g per week for women and < 210 g per week for men) and any other known specific cause of hepatic steatosis [3]. The prevalence in children/adolescents ranges from of 7.6% in general populations to 34.2% in obese populations [4]. Individuals with NAFLD are at elevated risk of several extrahepatic diseases, in particular type 2 diabetes mellitus and cardiovascular disease (CVD) [5], and have an increased all-cause mortality, with CVD as the leading cause of death [6].

NAFLD is associated with age, male gender, obesity [1], systemic insulin resistance [7] and dyslipidemia [8]. Furthermore, the heritability of hepatic fat accumulation and NAFLD has also been reported, where mutations in the patatin-like phospholipase domain-containing protein 3 (PNPLA3) gene and the transmembrane 6 superfamily 2 (TM6SF2) gene appear to play an important role [9–12].

Since the disease is increasingly diagnosed at younger age, it could have its origin in an early stage of life, possibly even *in utero* [13]. However, regarding maternal and perinatal risk factors, there is conflicting evidence on their independent influence on the development of NAFLD [14].

Thus, we describe maternal, neonatal, and adolescent risk factors for NAFLD in our 14- to 19-year-old Tyrolean cohort within the Early Vascular Ageing in the YOUth (EVA4YOU) study and examine their impact on the development of NAFLD in adolescence. To the best of our knowledge, we are the first to investigate an independent impact of risk predictors for NAFLD during different developmental periods in young people based on a detailed dataset.

## Methods

### Study participants

The EVA4YOU study is a single-center cross-sectional study, conducted between February 2021 and March 2023 at different schools and companies all over the county of Tyrol, Austria. The study assessed the health status of Tyrolean adolescents aged 14 to 19 years focusing on risk predictors and manifestations of early vascular ageing. Investigations within the study included a determination of the extent of fatty liver disease. For the current evaluation, all participants within the EVA4YOU study with a complete dataset for maternal and perinatal variables were included.

All participants provided a written informed consent, and if the participants were younger than 18 years, consent was additionally provided by their legal representatives. The study was approved by the ethics committee of the Medical University of Innsbruck (approval number: 1053/2020) and was conducted in accordance with the Declaration of Helsinki.

### Outcome parameter: Liver fat quantification

Liver fat content of the offspring was assessed by the controlled attenuation parameter (CAP) using signals acquired by FibroScan^®^ (Echosense, Paris, France), which is a standardized non-invasive measurement of hepatic steatosis [15]. For our adolescent cohort we used the FibroScan^®^ M-probe. Measurement was performed with the subject lying on the back with the right arm under the head and the right leg crossed over the left, causing a bend to the left side. The probe was positioned perpendicularly on the patient’s skin surface in an intercostal space along the medial axillary line using the xiphoid process as a reference point for determining an appropriate intercostal space over the center of the right hepatic lobe. Results were included in the final analysis only if all of the following criteria were met: fasting time of at least 3 hours, more than 10 complete measures, and the interquartile range < 30% of the median value. The device estimates hepatic steatosis in decibels/meter (dB/m) within the range of 100–400 dB/m. We used the 90^th^ percentile threshold for CAP values from a reference data set for defining manifest NAFLD [16] and CAP values as a continuous variable for further statistical analyses.

### Maternal factors

Maternal data were extracted from the mother-child booklet, the official Austrian pregnancy and early childhood medical record book. All study participants were asked to bring the booklet at the day of examination. The following maternal data were obtained: maternal weight and height before pregnancy, systolic and diastolic blood pressure before pregnancy and a history of maternal arterial hypertension, smoking status before/during pregnancy, age at pregnancy, abnormal oral glucose tolerance test during pregnancy or diabetes type 1 or 2 or gestational diabetes in history. Pre-pregnancy body mass index (BMI) was calculated as body weight before pregnancy in kilograms divided by the square of height before pregnancy in meters and it was categorized as underweight/normal (BMI < 25.0 kg/m^2^) and overweight/obese (BMI ≥ 25.0 kg/m^2^).

### Neonatal factors

Gestational age at birth and birth weight were extracted from the mother-child booklet. Birth weight z-scores were calculated using a reference data set [17]. Data regarding breastfeeding and breastfeeding duration were collected with an additional questionnaire distributed to the mothers. This questionnaire contained questions regarding the duration of exclusive breastfeeding and the duration of subsequent non-exclusive breastfeeding (months), or whether there was breastfeeding at all (yes/no). According to the national breastfeeding recommendations [18], a dichotomous variable for exclusive breastfeeding ≥ 4 months and < 4 months was created.

### Adolescent factors

#### Laboratory analysis

Blood samples were taken in the morning after an overnight fasting period, cooled and immediately delivered to the Central Institute of Medical and Chemical Laboratory Diagnostics of the Medical University of Innsbruck, Austria. Total cholesterol, low-density lipoprotein (LDL) cholesterol, high-density lipoprotein (HDL) cholesterol, triglycerides, gamma-glutamyltransferase, aspartate-transaminase, alanine-transaminase, and glucose were measured by enzymatic colorimetric assays. Lipoprotein(a), Ferritin and C-reactive protein were assessed with a particle-enhanced immunological clouding assay. Insulin and thyroid-stimulating hormone were determined with an electrochemiluminescence immunoassay (all with Cobas 8000, Roche Diagnostics, Rotkreuz, Switzerland). Glycated hemoglobin (HbA1c) was assessed by a high-pressure liquid chromatography (Tosoh G8, Tosoh Bioscience, Tessenderlo, Belgium) and total homocysteine by a chemiluminescence microparticle immunoassay (Architect, Abbott Laboratories, Abbott Park, Illinois, USA). Erythrocytes, hematocrit, and platelets were analyzed by an impedance method and leukocytes by biofluorescence/flow cytometry (all with XE-5000, Sysmex, Kobe, Japan). The Homeostatic Model Assessment for Insulin Resistance (HOMA-IR) index was calculated as fasting insulin (mU/l) multiplied by fasting glucose (mmol/L) divided by 22.5.

#### Assessment of lifestyle risk factors

Alcohol consumption, physical activity, and dietary patterns were assessed in a face-to-face interview, carried out by physicians. The product of alcohol content, volume, and intake frequency was used to calculate alcohol consumptions in grams per week. Physical activity was defined as moderate to vigorous sport at school or/and during leisure time in minutes per day. Regarding dietary patterns, a healthy diet score according to the recommendations of the American Heart Association for cardiovascular health in youth was obtained. In brief, it was composed of one point for each of the following components: ≥ 4.5 portions of fruits and vegetables per day, ≥ 2 portions of fish per week, ≥ 3 portions of fiber-rich nutriments per day, a salt-poor diet (< 1500 mg of salt per day) and consumption of ≤ 450 kcal of sugar-rich drinks per week (corresponds to ≤ 1 Liter of sugar-rich drinks per week) [19].

#### Socioeconomic status

Socioeconomic status was assessed using the Family Affluence Scale (FAS) III score, which was collected by a self-administered questionnaire. The score ranges from 0–13 points with a score of 0–7 being classified as low, of 8–11 as medium and 12–13 as high [20]. In this study, the FAS was used as a continuous variable (0–13).

#### Anthropometry

The physical examination included standardized measurements of body height and weight with calibrated scales. BMI was calculated as body weight in kilograms divided by the square of height in meters and converted into age and sex specific z-scores according to a German reference data set [21]. In addition, waist circumference was measured with a non-stretchable tape to the nearest 0.1 cm halfway between the iliac crest and the lower chest after a slight expiration. Z-scores were calculated using a German reference data set [22]. Systolic and diastolic blood pressure were calculated as the means of three measurements on the left and right arm, recorded after a 5-minute seated rest, using an automated oscillometric device (OMRON M4-I, Omron Healthcare Co., Lake Forest, Illinois, USA). Z-scores for blood pressures were calculated using a German reference data set [23].

### Statistical analysis

Characteristics of the study cohort are shown as count (percentage), mean ± SD, or median (interquartile range). Between-group differences were analyzed using χ^**2**^ test. Multivariable linear regression models with adjustment for sex and age were used to examine the association of each of the maternal, neonatal, and adolescent factors with CAP. The associations between sex and age with CAP and between maternal pre-pregnancy BMI and adolescent BMI were analyzed using simple linear regression. To determine the independent influence of adolescent factors on CAP, a multivariable linear regression model was performed including the adolescent factors HDL cholesterol, triglycerides, HOMA-IR, BMI z-score, and systolic blood pressure z-score (forced-entry method, model 1), with additional adjustment for birth weight for gestational age (model 2) and maternal pre-pregnancy BMI (model 3). Furthermore, mediation analysis with adjustment for sex and age was applied to assess the degree to which the association between maternal pre-pregnancy BMI and CAP is mediated by adolescent BMI. For all models, the assumptions (independence and normality of the errors, homoscedasticity, and linearity of the relationship between dependent and independent variables) were tested and satisfied and for all multivariable linear regression models collinearity was inspected by variance inflation factor (VIF). The analyses were conducted using SPSS version 29.0 (SPSS Inc., Chicago Illinois, USA) and R 4.3.1 (R Project for Statistical Computing, Vienna, Austria). Mediation analysis war performed using the PROCESS macro by Hayes (2022) in SPSS. P values were considered statistically significant at p < 0.05.

## Results

**Fig 1:**
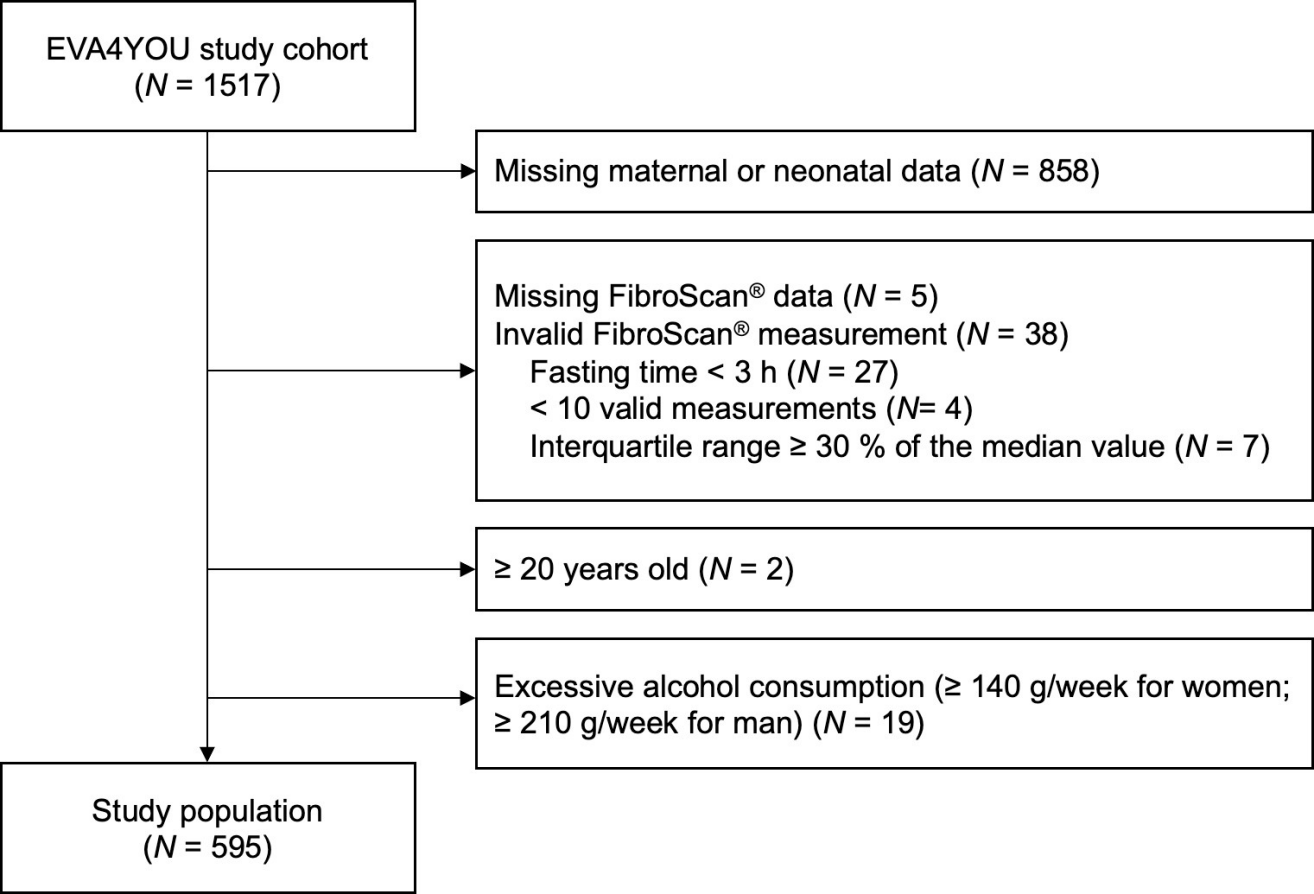
Study flow chart.

In total, 1517 adolescents were included in the EVA4YOU study. 659 participants had complete data on maternal and neonatal factors, of which 64 were excluded due to missing or invalid FibroScan^®^ measurements or excessive alcohol consumption (see flow chart in Fig 1). Therefore, our cohort consisted of 595 participants. In comparison to the EVA4YOU cohort, a higher proportion of female participants were included in the current study population (72.8% vs. 63.0%, p < 0.001), mainly because females were more likely to bring the mother-child booklet to the examination and participants without data from the mother-child booklet were excluded from this analysis. For each sex individually, characteristics were very similar as in the full study cohort (see also S1 Table).

Mean age of our study cohort was 17.0 ± 1.3 years, and 433 participants (72.8%) were female. Detailed characteristics of the study population are shown in Table 1. Using the 90^th^ percentile threshold from a reference data set, 29 participants (4.9%) of our cohort had manifest NAFLD.

**Table 1:** Characteristics of the study population.

	All *N* = 595 (100%)	Males *N* = 162 (27.2%)	Females *N* = 433 (72.8%)
**Maternal factors**			
BMI before pregnancy, kg/m^2^	22.3 (20.6–25.0)		
BMI ≥25 kg/m^2^	145 (24.4%)		
SBP before pregnancy, mmHg	116 ± 14		
DBP before pregnancy, mmHg	70 ± 10		
Arterial hypertension before pregnancy^a^	56 (9.4%)		
Smoking before pregnancy	86 (16.1%)		
1–10 cigarettes per day	60 (11.2%)		
>10 cigarettes per day	26 (4.9%)		
Age during pregnancy, years	29.8 ± 5.2		
Abnormal oGTT or diabetes mellitus I/II	16 (2.7%)		
**Neonatal factors**			
Gestational age at birth, weeks	39.4 (38.4–40.3)	39.5 (38.3–40.7)	39.4 (38.4–40.3)
Birth weight, kg	3.23 ± 0.52	3.30 ± 0.56	3.20 ± 0.50
Birth weight for gestational age, z-score	-0.097 ± 1.045	-0.204 ± 0.860	-0.058 ± 1.104
Duration of exclusive and non-exclusive breastfeeding, months	6.0 (2.0–10.1)	6.0 (2.5–11.8)	6.0 (2.0–9.0)
Exclusive breastfeeding for ≥4 months	281 (57.2%)	65 (55.6%)	216 (57.8%)
**Adolescent factors**			
Age, years	17.0 ± 1.3	16.9 ± 1.2	17.1 ± 1.3
CAP, dB/m	183.1 ± 41.5	197.2 ± 35.1	177.8 ± 42.4
CAP ≥90^th^ percentile^b^	29 (4.9%)	12 (7.4%)	17 (3.9%)
Alanine transaminase, U/L	15.0 (12.0–20.0)	18.0 (15.0–23.0)	14.0 (12.0–18.0)
Aspartate transaminase, U/L	21.0 (18.0–25.0)	24.0 (20.0–28.0)	20.0 (17.0–23.0)
Gamma-glutamyltransferase, U/L	13.0 (11.0–17.0)	17.0 (13.0–21.0)	13.0 (10.0–16.0)
Total cholesterol, mg/dl	159.3 ± 28.3	142.8 ± 20.6	165.5 ± 28.4
HDL cholesterol, mg/dl	58.0 ± 13.1	50.7 ± 9.0	60.8 ± 13.4
LDL cholesterol, mg/dl	89.5 ± 22.9	81.6 ± 19.0	92.4 ± 23.6
Non-HDL cholesterol, mg/dl	101.7 ± 25.8	91.5 ± 21.4	104.8 ± 26.2
Triglycerides, mg/dl	71.0 (57.0–100.0)	69.0 (54.0–93.0)	72.0 (58.0–102.0)
Lipoprotein(a), nmol/l	36.9 ± 62.9	26.6 ± 52.9	40.7 ± 65.9
Total homocysteine, μmol/l	10.2 (8.6–12.2)	10.9 (9.1–12.7)	9.9 (8.5–11.3)
Fasting glucose, mg/dl	75.0 ± 10.2	78.3 ± 10.1	73.8 ± 9.9
HbA1c,%	5.3 ± 0.3	5.3 ± 0.3	5.3 ± 0.3
Insulin, mU/l	12.6 (9.2–16.5)	12.0 (8.8–16.2)	12.8 (9.6–16.5)
HOMA-IR, mlU×mmol	2.3 (1.6–3.1)	2.3 (1.6–3.3)	2.3 (1.6–3.1)
C-reactive protein, mg/dl	0.14 ± 0.34	0.10 ± 0.35	0.16 ± 0.33
TSH, mU/l	2.1 (1.6–2.8)	2.2 (1.6–3.1)	2.0 (1.5–2.6)
Ferritin, μg/l	41.0 (25.3–68.5)	69.0 (43.0–104.0)	35.0 (21.5–56.0)
Hematocrit, l/l	0.43 ± 0.03	0.46 ± 0.03	0.41 ± 0.02
Erythrocytes, T/l	4.9 ± 0.4	5.4 ± 0.4	4.7 ± 0.3
Leucocytes, G/l	6.3 ± 1.7	5.8 ± 1.4	6.5 ± 1.7
Thrombocytes, G/l	287.3 ± 61.2	256.5 ± 50.4	298.9 ± 61.0
Alcohol intake, g/week	28.2 (12.5–57.1)	50.0 (11.0–96.7)	25.3 (12.5–50.5)
Physical activity, min/day	64.9 ± 54.9	82.7 ± 62.7	58.2 ± 50.1
Healthy diet score	1.0 (1.0–1.0)	1.0 (0.0–1.0)	1.0 (1.0–1.0)
Family Affluence Scale score	9.5 ± 1.7	9.5 ± 1.8	9.5 ± 1.7
BMI, kg/m^2^	21.5 (19.6–23.8)	21.7 (19.6–24.3)	21.3 (19.6–23.6)
BMI, z-score	-0.088 ± 1.005	-0.015 ± 0.997	-0.116 ± 1.008
Waist circumference, cm	71.0 (66.0–78.0)	77.0 (72.0–83.0)	69.0 (65.0–75.0)
Waist circumference, z-score	0.046 ±1.128	0.238 ± 1.000	-0.026 ± 1.166
SBP, mmHg	127 ± 12	132 ± 11	125 ± 11
SBP, z-score	1.104 ± 1.091	0.936 ± 0.958	1.166 ± 1.133
DBP, mmHg	75 ± 8	72 ± 7	76 ± 8
DBP, z-score	0.605 ± 1.078	0.129 ± 0.941	0.784 ± 1.072

Values are given as mean ± SD, median (interquartile range), or count (%).

BMI, body mass index; SBP, systolic blood pressure; DBP, diastolic blood pressure; oGTT, oral glucose tolerance testing; CAP, controlled attenuation parameter; HDL, high-density lipoprotein; LDL, low-density lipoprotein; HbA1c, glycated hemoglobin; HOMA-IR, Homeostatic Model Assessment for Insulin Resistance; and TSH, thyroid-stimulating hormone.

Missing values were <2% except for smoking status before pregnancy (10.1%), breastfeeding data (17.5%), non-HDL cholesterol (8.1%), lipoprotein(a) (2.5%), total homocysteine (3.2%), Insulin and HOMA-IR (both 5.2%).

^a^ SBP before pregnancy ≥ 140 mmHg, DBP before pregnancy ≥ 90 mmHg, or arterial hypertension in history.

^b^ Threshold for manifest non-alcoholic fatty liver disease; calculated using a reference data set [16].

**Fig 2:**
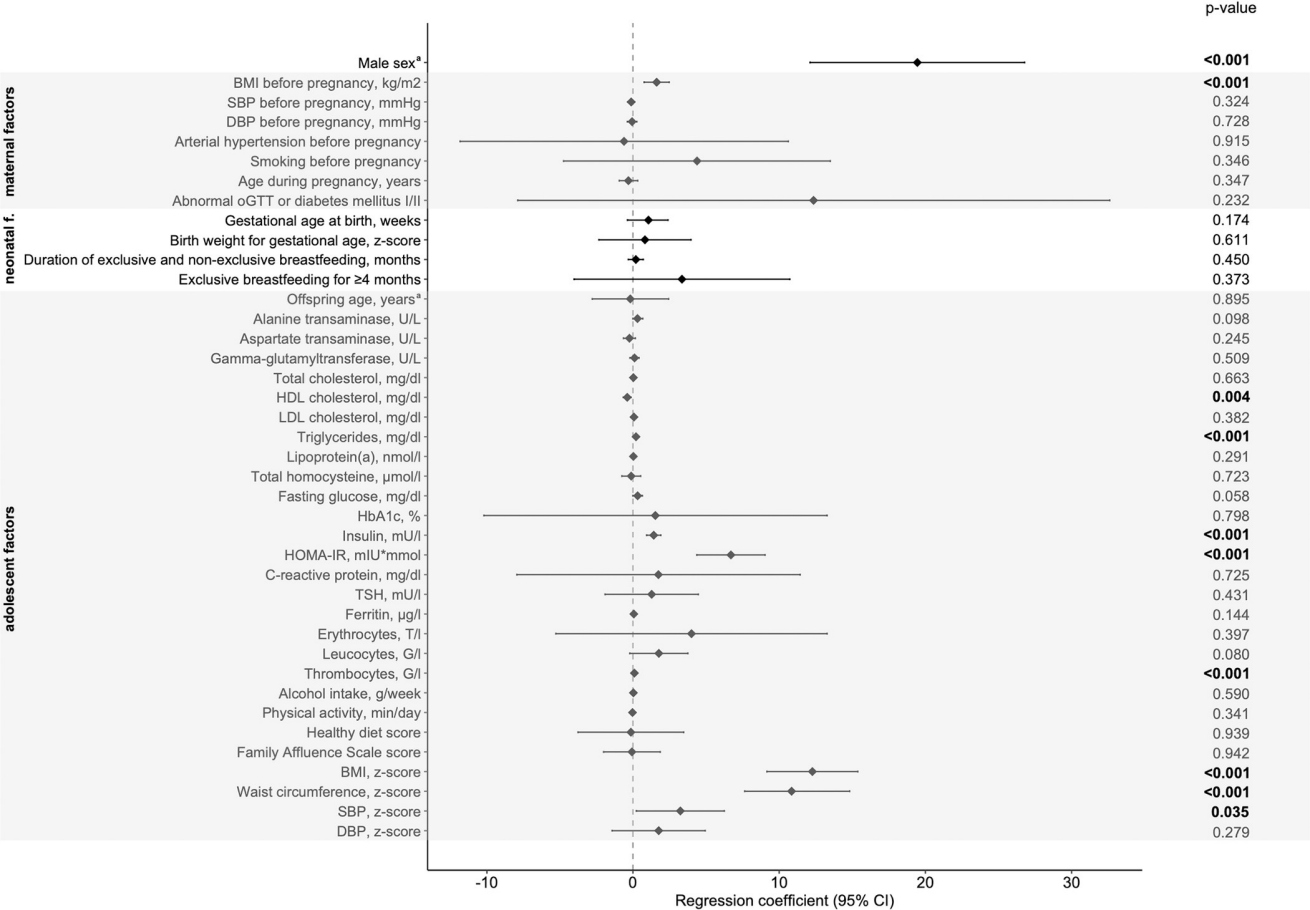
Association of maternal, neonatal, and adolescent factors with CAP values. Linear regression with adjustment for sex and age (if not otherwise specified). Factors are shown on the y-axis and regression coefficients are shown on the x-axis as points with the corresponding 95% confidence intervals as horizontal segments. BMI, body mass index; SBP, systolic blood pressure; DBP, diastolic blood pressure; oGTT, oral glucose tolerance testing; HDL, high-density lipoprotein; LDL, low-density lipoprotein; HbA1c, glycated hemoglobin; HOMA-IR, Homeostatic Model Assessment for Insulin Resistance; and TSH, thyroid-stimulating hormone. ^a^ Analyzed with unadjusted linear regression.

**Fig 3:**
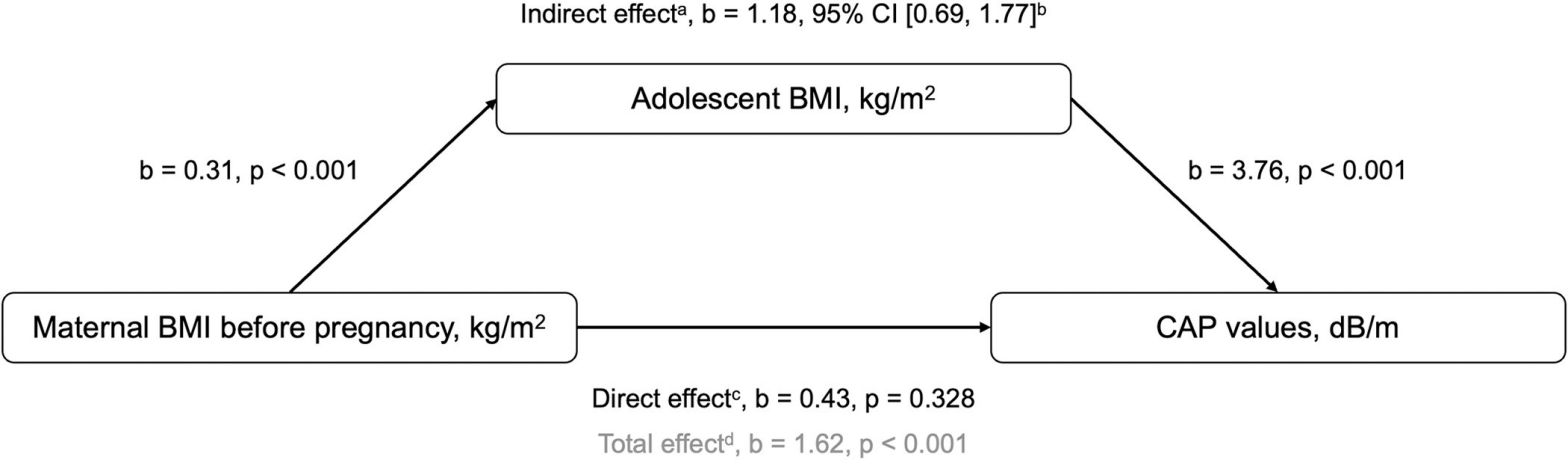
Mediation analysis model. Maternal pre-pregnancy BMI as a predictor of adolescent CAP values, mediated by adolescent BMI. The confidence interval for the indirect effect is a BCa bootstrapped CI based on 5000 samples. The model was adjusted for offspring sex and age. CI, confidence interval; BMI, body mass index; and CAP, controlled attenuation parameter. ^a^ Indirect effect: the effect of maternal pre-pregnancy BMI on CAP values acting through the mediator (adolescent BMI). ^b^ Statistically significant. ^c^ Direct effect: the effect of maternal pre-pregnancy BMI on CAP values, not explained by the mediator.^d^ Total effect: the effect of maternal pre-pregnancy BMI on CAP values (no mediator in the model).

Male sex had a significant influence on higher CAP values in all models (p < 0.001, each). Regarding *maternal factors* there was a significant association between maternal pre-pregnancy BMI and CAP in the linear regression model after adjustment for sex and age (Fig 2) and this association remained significant after adjustment for birth weight for gestational age (p < 0.001, each). Due to the highly significant correlation between maternal pre-pregnancy BMI and adolescent BMI in our cohort (b = 0.31, p < 0.001), a mediation analysis was performed to determine the mediation effect of adolescent BMI on the association between maternal pre-pregnancy BMI and adolescent CAP values (Fig 3). In this analysis, the significant direct association of maternal pre-pregnancy BMI and CAP (total effect b = 1.62, p < 0.001) was rendered non-significant after including the mediator in the model (direct effect b = 0.43, p = 0.328). The relationship between maternal pre-pregnancy BMI and adolescent CAP values was predominantly mediated by adolescent BMI (indirect effect b = 1.18, 95% CI [0.69, 1.77]). *Neonatal factors* (gestational age at birth, birth weight for gestational age z-score, breastfeeding duration) were not related to CAP values in adolescence. *Adolescent factors* such as triglyceride and insulin levels, the HOMA-IR index, platelet count, BMI z-score and waist circumference z-score (p < 0.001, each), as well as HDL cholesterol levels (p = 0.004), and systolic blood pressure z-score (p = 0.035) showed a significant association with CAP values (Fig 2). However, in the multivariable analysis which included these adolescent factors, only triglyceride levels (p = 0.012), HOMA-IR index (p < 0.001), and BMI z-score (p < 0.001) remained significantly associated with CAP values (model 1, Table 2), and remained highly significant after adjustment for birth weight for gestational age (model 2, Table 2) and maternal pre-pregnancy BMI (model 3, Table 2). There was no significant impact of healthy diet score, physical activity, or socioeconomic status on CAP values.

**Table 2:** Multivariable association between adolescent factors and CAP values.

	Model 1 (R^2^ = 0.160)		Model 2 (R^2^ = 0.164)		Model 3 (R^2^ = 0.167)	
	Regression coefficient (95% CI)	P value	Regression coefficient (95% CI)	P value	Regression coefficient (95% CI)	P value
**Adolescent factors**						
HDL cholesterol, mg/dl	-0.11 (-0.37, 0.15)	0.418	-0.13 (-0.39, 0.13)	0.327	-0.13 (-0.40, 0.13)	0.316
Triglycerides, mg/dl	0.12 (0.03, 0.21)	**0.012**	0.11 (0.12, 0.20)	**0.021**	0.11 (0.02, 0.20)	**0.021**
HOMA-IR, mlU×mmol	4.26 (1.84, 6.67)	**<0.001**	5.09 (2.52, 7.65)	**<0.001**	5.12 (2.55, 7.69)	**<0.001**
BMI, z-score	9.66 (6.26, 13.05)	**<0.001**	8.95 (5.49, 12.42)	**<0.001**	8.23 (4.60, 11.85)	**<0.001**
SBP, z-score	-2.28 (-5.31, 0.76)	0.141	-1.99 (-5.04, 1.06)	0.200	-2.01 (-5.05, 1.04)	0.196
**Neonatal factor**						
Birth weight for gestational age, z-score			1.34 (-1.89, 4.57)	0.417	0.97 (-2.30, 4.24)	0.559
**Maternal factor**						
BMI before pregnancy, kg/m^2^					0.60 (-0.29, 1.49)	0.188

Multivariable linear regression (forced-entry method). All models are adjusted for sex and age. Male sex had a significant influence on higher CAP values in all models (p < 0.001, each).

CI, confidence interval; HDL, high-density lipoprotein; HOMA-IR, Homeostatic Model Assessment for Insulin Resistance; BMI, body mass index; and SBP, systolic blood pressure.

## Discussion

In the current study, we investigated the independent impact of maternal, neonatal, and adolescent risk factors on the development of NAFLD in adolescents and could show that higher CAP values in the young were mainly driven by adolescent risk predictors of the metabolic syndrome.

NAFLD is the most common form of chronic liver disease and a prevalent metabolic condition even in adolescents. In our study cohort, 4.9% were classified as having manifest NAFLD, which is slightly lower than the prevalence of NAFLD in adolescents from general population studies estimated in a large meta-analysis at 7.6% [4]. The difference in prevalence might be due to the heterogeneity in methods of diagnosing NAFLD. In 56% of the studies included in the meta-analysis by Anderson et al. NAFLD was diagnosed by ultrasound and in further 44% by elevated alanine-transaminase [4], whereas in our study quantification of steatosis was analyzed by CAP (FibroScan^®^ M-probe) using a threshold for CAP values from a reference data set [16]. A lower proportion of included male participants in the current study may also have contributed to lower prevalence of NAFLD, because, compared to females, males show a higher prevalence of NAFLD [24]. Also in our study, the prevalence of manifest NAFLD showed a strong tendency to be higher in males than in females (7.4% vs. 3.9%, p = 0.089) and male sex had a significant association with increased CAP values in all statistical models. This association might be due to the protective functions of estrogens in females [25]. It has been shown that hepatic estrogen signaling strongly contributes to sex differences in the regulation of hepatic metabolism [26] by conferring the ability to adapt the metabolic response to an excess of dietary lipids to females but not to males [27].

### NAFLD and maternal factors

Maternal pre-pregnancy BMI was significantly associated with adolescent CAP values in our cohort after adjustment for sex, age, and birth weight for gestational age. This finding is consistent with previous cross-sectional and cohort studies, although those studies did not consider the influence of neonatal factors on this association [28–33]. The relation between maternal factors and adolescent liver fat content led to the hypothesis that NAFLD may have its origin already *in utero* [13]. Even in animal models, maternal obesity during pregnancy predisposes the developing offspring to NAFLD and insulin resistance [34, 35]. However, we were able to show that the influence of maternal pre-pregnancy BMI on CAP values was predominantly mediated by adolescent BMI. Our analysis therefore suggest that increased maternal pre-pregnancy BMI leads to NAFLD in the offspring, but this is mainly due to increased adolescent BMI. The association between material pre-pregnancy BMI and adolescent BMI might be explained by direct intrauterine mechanisms as well as environmental, lifestyle, or genetic characteristics [36, 37].

We could not find any association of liver fat content of the offspring with all other maternal factors like arterial hypertension, smoking at/during pregnancy, and maternal diabetes. However, there are conflicting results in literature regarding maternal diabetes and liver fat content with some studies reporting an association [38] and others who could not corroborate these findings [39, 40].

### NAFLD and neonatal factors

All neonatal factors evaluated had no effect on CAP values in our cohort. Likewise, no association between birth weight and NAFLD was found in other studies [41–44]. Regarding the association between liver fat accumulation and being born preterm, there is conflicting evidence in the literature: some studies reported higher liver fat content or prevalence of NAFLD in young people born < 34 weeks [45], whereas another study did not confirm this finding [46]. Breastfeeding duration was not associated with CAP values in our analysis, neither was maintenance of the national breastfeeding recommendation of at least 4 months of exclusive breastfeeding [18]. In contrast, Ayonrinde et al. [29] and Rajindrajith et al. [47] reported a significant reduction in NAFLD with breastfeeding duration, whereas other studies did not find this association [28, 32].

### NAFLD and adolescent factors

In the current study adolescent factors such as triglyceride levels, the HOMA-IR index, and BMI z-score were significantly and independently associated with an increased risk of NAFLD in the multivariable regression model after adjustment for neonatal and maternal factors. Obesity is one of the main risk factors for NAFLD in adolescence [1] and the discrepancy in the prevalence of NAFLD between normal-weight and overweight adolescents (7.6% vs. 34.2%) clearly illustrates this relationship [4]. The independent association of elevated serum triglyceride levels with hepatic steatosis, which has also been described in previous literature [48], can be explained by the fact that fat accumulates in the liver of patients with NAFLD mainly in the form of triglycerides [49, 50]. In addition, insulin resistance, which is mainly caused by dietary and environmental factors as well as obesity, is one of the key factors in the development of hepatic steatosis. It increases hepatic de novo lipogenesis and impairs the inhibition of adipose tissue lipolysis, resulting in an increased flux of fatty acids to the liver [51]. NAFLD is increasingly recognized as the liver component of the metabolic syndrome [3], and therefore it is not surprising that these two disease patterns are associated with the same risk factors.

### Strengths and limitations

A strength of our study is the broad dataset including maternal, neonatal, and adolescent factors of a large, homogenous, and well-characterized study cohort. Furthermore, all determinants of NAFLD were collected per protocol and examinations and face-to-face interviews were performed by experienced and trained personnel. Information regarding maternal data, gestational age, and birth weight was taken from mother-child booklets, which are official Austrian pregnancy and early childhood medical record books and data are very reliable.

Breastfeeding information was collected by a questionnaire years after the breastfeeding period and recall bias might be present. As a further limitation, it must be noted that 922 adolescents had to be excluded from our analysis mainly because of the unavailability of mother-child booklets and missing maternal and neonatal data. Therefore, selection bias cannot be excluded. In addition, it must be mentioned that more females than males were included in the current study cohort. However, because we adjusted all our models for sex, our results can be considered representative. A further limitation is that we could not distinguish between NAFLD and non-alcoholic steatohepatitis (NASH) by using CAP measurements, but liver biopsy, which is the gold standard to distinguish steatosis from NASH is not justifiable in a large cohort of asymptomatic adolescents.

## Conclusion

Maternal pre-pregnancy BMI predicted adolescent liver fat content, but the association is predominantly mediated by adolescent BMI. Furthermore, neonatal factors probably do not contribute to the development of hepatic steatosis. We therefore conclude that only adolescent risk factors have a direct influence on the degree of fatty liver disease in adolescents and support the concept that NAFLD represents the liver manifestation of the metabolic syndrome.

Detection of NAFLD in adolescents is crucial because the extent of the disease is amenable to lifestyle modification, and early intervention regarding risk factors for fatty liver disease may therefore be important.

## Supporting information

S1 Checklist STROBE statement—checklist of items that should be included in reports of cross-sectional studies. (DOC)

S1 Table Characteristics of the EVA4YOU cohort. (DOCX)

S1 Dataset Deidentified original dataset. (XLSX)
