# Supplementary material for: Determinants of non-alcoholic fatty liver disease in young people: Maternal, neonatal, and adolescent factors
Source: PLoS One. 2024 Feb 22;19(2):e0298800. doi: 10.1371/journal.pone.0298800 (PMC10883560; doi:10.1371/journal.pone.0298800)
Supplement: S1 Table — (DOCX) [file pone.0298800.s002.docx]

S1 Table. Characteristics of the EVA4YOU cohort

|  | Study cohort  *N* = 595 | |  | EVA4YOU cohort  *N* = 1517 | |
| --- | --- | --- | --- | --- | --- |
|  | Males  *N* = 162 (27.2%) | Females  *N* = 433 (72.8%) |  | Males  *N* = 561 (37.0%) | Females  *N* = 956 (63.0%) |
| Age, years | 16.9 ± 1.2 | 17.1 ± 1.3 |  | 17.3 ± 1.6 | 17.2 ± 1.3 |
| CAP, dB/m | 197.2 ± 35.1 | 177.8 ± 42.4 |  | 198.3 ± 40.4 | 179.9 ± 41.3 |
| Alanine transaminase, U/L | 18.0 (15.0-23.0) | 14.0 (12.0-18.0) |  | 19.0 (15.8-26.0) | 14.0 (12.0-18.0) |
| Aspartate transaminase, U/L | 24.0 (20.0-28.0) | 20.0 (17.0-23.0) |  | 24.0 (20.0-29.0) | 20.0 (17.0-23.0) |
| Gamma-glutamyltransferase, U/L | 17.0 (13.0-21.0) | 13.0 (10.0-16.0) |  | 17.0 (13.0-21.3) | 13.0 (10.0-16.0) |
| Total cholesterol, mg/dl | 142.8 ± 20.6 | 165.5 ± 28.4 |  | 147.9 ± 24.8 | 164.7 ± 29.2 |
| HDL cholesterol, mg/dl | 50.7 ± 9.0 | 60.8 ± 13.4 |  | 51.0 ± 10.3 | 60.2 ± 13.3 |
| LDL cholesterol, mg/dl | 81.6 ± 19.0 | 92.4 ± 23.6 |  | 85.2 ± 22.7 | 91.9 ± 23.9 |
| Triglycerides, mg/dl | 69.0 (54.0-93.0) | 72.0 (58.0-102.0) |  | 74.0 (56.0-99.3) | 76.0 (57.0-103.0) |
| Lipoprotein(a), nmol/l | 26.6 ± 52.9 | 40.7 ± 65.9 |  | 31.8 ± 62.9 | 39.5 ± 67.2 |
| Total homocysteine, μmol/l | 10.9 (9.1-12.7) | 9.9 (8.5-11.3) |  | 10.5 (9.0-12.6) | 9.9 (8.5-11.6) |
| Fasting glucose, mg/dl | 78.3 ± 10.1 | 73.8 ± 9.9 |  | 80.3 ± 11.4 | 74.3 ± 9.5 |
| HbA1c, % | 5.3 ± 0.3 | 5.3 ± 0.3 |  | 5.2 ± 0.3 | 5.2 ± 0.3 |
| Insulin, mU/l | 12.0 (8.8-16.2) | 12.8 (9.6-16.5) |  | 12.2 (8.8-16.2) | 12.6 (9.3-16.5) |
| HOMA-IR, mlU×mmol | 2.3 (1.6-3.3) | 2.3 (1.6-3.1) |  | 2.3 (1.7-3.3) | 2.3 (1.6-3.1) |
| C-reactive protein, mg/dl | 0.10 ± 0.35 | 0.16 ± 0.33 |  | 0.11 ± 0.33 | 0.17 ± 0.35 |
| TSH, mU/l | 2.2 (1.6-3.1) | 2.0 (1.5-2.6) |  | 2.1 (1.6-2.8) | 2.1 (1.5-2.7) |
| Ferritin, μg/l | 69.0 (43.0-104.0) | 35.0 (21.5-56.0) |  | 78.0 (51.0-112.3) | 36.0 (22.0-58.0) |
| Erythrocytes, T/l | 5.4 ± 0.4 | 4.7 ± 0.3 |  | 5.3 ± 0.4 | 4.7 ± 0.3 |
| Leucocytes, G/l | 5.8 ± 1.4 | 6.5 ± 1.7 |  | 5.9 ± 1.5 | 6.6 ± 1.7 |
| Thrombocytes, G/l | 256.5 ± 50.4 | 298.9 ± 61.0 |  | 260.7 ± 52.2 | 299.6 ± 61.5 |
| Alcohol intake, g/week | 50.0 (11.0-96.7) | 25.3 (12.5-50.5) |  | 50.0 (9.6-106.4) | 28.2 (12.5-56.4) |
| Physical activity, min/day | 82.7 ± 62.7 | 58.2 ± 50.1 |  | 100.1 ± 90.4 | 57.6 ± 51.6 |
| Healthy diet score | 1.0 (0.0-1.0) | 1.0 (1.0-1.0) |  | 1.0 (0.0-1.0) | 1.0 (1.0-1.0) |
| Family Affluence Scale score | 9.5 ± 1.8 | 9.5 ± 1.7 |  | 9.3 ± 1.9 | 9.5 ± 1.8 |
| BMI, z-score | -0.015 ± 0.997 | -0.116 ± 1.008 |  | 0.069 ± 0.997 | -0.101 ± 1.063 |
| Waist circumference, z-score | 0.238 ± 1.000 | -0.026 ± 1.166 |  | 0.328 ± 0.990 | 0.016 ± 1.177 |
| SBP, z-score | 0.936 ± 0.958 | 1.166 ± 1.133 |  | 0.813 ± 0.976 | 1.053 ± 1.114 |
| DBP, z-score | 0.129 ± 0.941 | 0.784 ± 1.072 |  | 0.166 ± 0.957 | 0.742 ± 1.053 |

Values are given as mean ± SD, median (interquartile range), or count (%).

HDL, high-density lipoprotein; LDL, low-density lipoprotein; HbA1c, glycated hemoglobin; HOMA-IR, Homeostatic Model Assessment for Insulin Resistance; TSH, thyroid-stimulating hormone; BMI, body mass index; SBP, systolic blood pressure; and DBP, diastolic blood pressure.
